# (*S*)-(+)-4-(Oxiran-2-ylmeth­oxy)-9*H*-carbazole

**DOI:** 10.1107/S1600536810038900

**Published:** 2010-10-09

**Authors:** Ding-Qiang Lu, Jia Chen, Wen-Yuan Wu, Xiu-Quan Ling, Ya-Jun Chang

**Affiliations:** a College of Pharmaceutical Sciences, Nanjing University of Technolgy, Nanjing 210009, People’s Republic of China; b College of Science, Nanjing University of Technolgy, Nanjing 210009, People’s Republic of China

## Abstract

In the title compound, C_15_H_13_NO_2_, all atoms of the carbazole group are coplanar (r.m.s. deviation = 0.005 Å), and the dihedral angle between this plane and C—O—C plane of oxane group is 57.1 (4)°. The crystal packing is stabilized by an N—H⋯O hydrogen bond, resulting in infinite supra­molecular chains along [001].

## Related literature

For general background to the target product, see: Hildesheim *et al.* (2002 ▶); Morgan (1994 ▶). For other inter­mediates with similar structures, see: Herbert *et al.* (1987 ▶). For assignment of the absolute structure based on the synthesis, see: Rao *et al.* (2007 ▶)
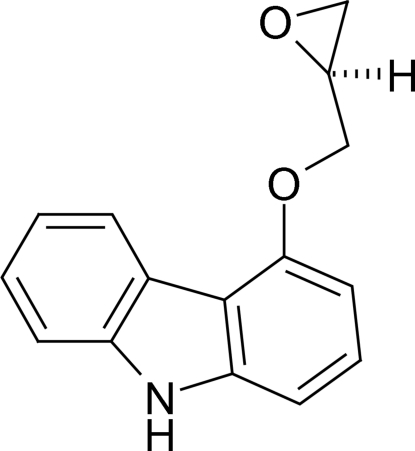


## Experimental

### 

#### Crystal data

C_15_H_13_NO_2_
*M* *_r_* = 239.26
Orthorhombic, 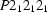

*a* = 7.6140 (15) Å
*b* = 9.5870 (19) Å
*c* = 16.628 (3) Å
*V* = 1213.8 (4) Å^3^
*Z* = 4
Mo *K*α radiation
μ = 0.09 mm^−1^
*T* = 293 K
0.30 × 0.10 × 0.10 mm

#### Data collection

Enraf–Nonius CAD-4 diffractometer
Absorption correction: ψ scan (North *et al.*, 1968 ▶) *T*_min_ = 0.974, *T*_max_ = 0.991
2198 measured reflections
1298 independent reflections
834 reflections with *I* > 2σ(*I*)
*R*_int_ = 0.061
3 standard reflections every 200 reflections  intensity decay: none

#### Refinement

*R*[*F* ^2^ > 2σ(*F* ^2^)] = 0.058
*wR*(*F* ^2^) = 0.124
*S* = 1.05
1298 reflections
163 parameters
H-atom parameters constrained
Δρ_max_ = 0.18 e Å^−3^
Δρ_min_ = −0.17 e Å^−3^

### 

Data collection: *CAD-4 Software* (Enraf–Nonius, 1989 ▶); cell refinement: *CAD-4 Software*; data reduction: *XCAD4* (Harms & Wocadlo, 1995 ▶); program(s) used to solve structure: *SHELXTL* (Sheldrick, 2008 ▶); program(s) used to refine structure: *SHELXTL*; molecular graphics: *SHELXTL*; software used to prepare material for publication: *SHELXTL*.

## Supplementary Material

Crystal structure: contains datablocks I, global. DOI: 10.1107/S1600536810038900/fl2309sup1.cif

Structure factors: contains datablocks I. DOI: 10.1107/S1600536810038900/fl2309Isup2.hkl

Additional supplementary materials:  crystallographic information; 3D view; checkCIF report

## Figures and Tables

**Table 1:** Hydrogen-bond geometry (Å, °)

*D*—H⋯*A*	*D*—H	H⋯*A*	*D*⋯*A*	*D*—H⋯*A*
N1—H1⋯O2^i^	0.86	2.09	2.948 (5)	172

Symmetry code: (i) 
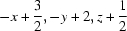
.

**Fig. 1.**
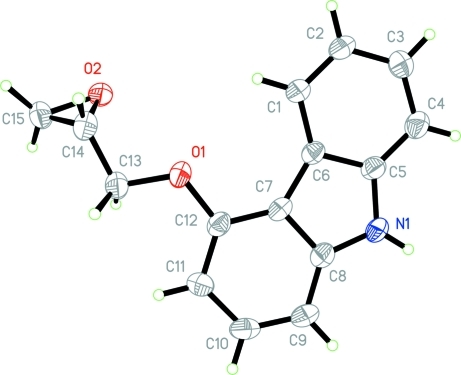
The molecular structure of (I), with atom labels and 30% probability displacement ellipsoids for non-H atoms.

**Fig. 2.**
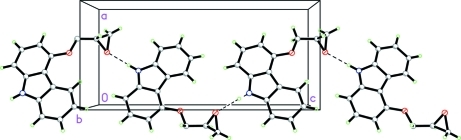
Supramolecular chains along the [010] direction by N–H···O hydrogen bonds (dashed lines).

## supplementary crystallographic information

### Comment

4-(2,3-epoxypropoxy)carbazole is used as a starting agent for the synthesis of 1-(carbazol-4-yloxy-3-[[2-(*O*-methoxyphenoxy)ethyl]amino]-2-propranol (Herbert & Heppenheim, 1987; Hildesheim *et al.*, 2002), which is a commercial drug (carvedilol) with α- and β_1_– receptor blocking activity that has been approved for the treatment of congestive heart failure (CHF). However carvedilol is actually a racemic mixture of the *R* and  S enantiomers, and the β-receptor blocking activity of the S-enantiomer is about 200 times higher than that of *R*-carvedilol (Morgan, 1994).

We have now synthesized the title compound (CAS:67843–74-7), (I), as an intermediate in the synthesis of the target molecule, S-carvedilol, and report its structure here. The optically pure (*R*)-(-)-epichlorohydrin (CAS: 51594–55-9) was used as the starting agent, and during the reaction, an inversion of the chiral C atom occurred to give the final product (I) (Rao *et al.*, 2007).

Both the carbazole group and oxane group are planar, and the dihedral angle between them is 57.1 (4). The molecules are stacked along the *a* axis, and linked by N–H..O hydrogen bonds to form infinite chains along the [001] direction,

### Experimental

For the preparation of the title compound, K_2_CO_3_ (20.73 g, 0.15 mol) and (*R*)-(-)-epichlorohydrin (7 ml, 0.09 mol) were added to an IPA (60 ml) solution containing 4-hydroxycarbazole (10.98 g, 0.06 mol). Then the reaction mixture was refluxed for 5 h at 355 K. The crude product was purified by recrystallization from ethyl acetate to provide colourless crystals suitable for X-ray analysis.

### Refinement

H atoms were positioned geometrically [N–H = 0.86 Å, and C–H = 0.93, 0.97 and 0.98 Å for aromatic, methyne and methine H atoms, respectively] and constrained to ride on their parent atoms, with *U*_iso_(H) = 1.2*U*_eq_(C or N). In the absence of significant anomalous scattering effects, Friedel pairs were merged for the final cycles of refinement.

### Crystal data

**Table:**

C_15_H_13_NO_2_	*F*(000) = 504
*M**_r_* = 239.26	*D*_x_ = 1.309 Mg m^−^^3^
Orthorhombic, *P*2_1_2_1_2_1_	Mo *K*α radiation, λ = 0.71073 Å
Hall symbol: P 2ac 2ab	Cell parameters from 25 reflections
*a* = 7.6140 (15) Å	θ = 9–13°
*b* = 9.5870 (19) Å	µ = 0.09 mm^−^^1^
*c* = 16.628 (3) Å	*T* = 293 K
*V* = 1213.8 (4) Å^3^	Prism, colourless
*Z* = 4	0.30 × 0.10 × 0.10 mm

### Data collection

**Table:**

Enraf–Nonius CAD-4 diffractometer	834 reflections with *I* > 2σ(*I*)
Radiation source: fine-focus sealed tube	*R*_int_ = 0.061
graphite	θ_max_ = 25.4°, θ_min_ = 2.5°
ω/2θ scans	*h* = −9→9
Absorption correction: ψ scan (North *et al.*, 1968)	*k* = 0→11
*T*_min_ = 0.974, *T*_max_ = 0.991	*l* = 0→19
2198 measured reflections	3 standard reflections every 200 reflections
1298 independent reflections	intensity decay: none

### Refinement

**Table:**

Refinement on *F*^2^	Primary atom site location: structure-invariant direct methods
Least-squares matrix: full	Secondary atom site location: difference Fourier map
*R*[*F*^2^ > 2σ(*F*^2^)] = 0.058	Hydrogen site location: inferred from neighbouring sites
*wR*(*F*^2^) = 0.124	H-atom parameters constrained
*S* = 1.05	*w* = 1/[σ^2^(*F*_o_^2^) + (0.0341*P*)^2^ + 0.3329*P*] where *P* = (*F*_o_^2^ + 2*F*_c_^2^)/3
1298 reflections	(Δ/σ)_max_ < 0.001
163 parameters	Δρ_max_ = 0.18 e Å^−^^3^
0 restraints	Δρ_min_ = −0.16 e Å^−^^3^

### Special details

**Table:**

Geometry. All e.s.d.'s (except the e.s.d. in the dihedral angle between two l.s. planes) are estimated using the full covariance matrix. The cell e.s.d.'s are taken into account individually in the estimation of e.s.d.'s in distances, angles and torsion angles; correlations between e.s.d.'s in cell parameters are only used when they are defined by crystal symmetry. An approximate (isotropic) treatment of cell e.s.d.'s is used for estimating e.s.d.'s involving l.s. planes.
Refinement. Refinement of *F*^2^ against ALL reflections. The weighted *R*-factor *wR* and goodness of fit *S* are based on *F*^2^, conventional *R*-factors *R* are based on *F*, with *F* set to zero for negative *F*^2^. The threshold expression of *F*^2^ > σ(*F*^2^) is used only for calculating *R*-factors(gt) *etc*. and is not relevant to the choice of reflections for refinement. *R*-factors based on *F*^2^ are statistically about twice as large as those based on *F*, and *R*- factors based on ALL data will be even larger.

### Fractional atomic coordinates and isotropic or equivalent isotropic displacement parameters (Å^2^)

**Table:**

	*x*	*y*	*z*	*U*_iso_*/*U*_eq_	
N1	0.8591 (5)	1.1011 (4)	0.7894 (2)	0.0555 (11)	
H1	0.9127	1.1158	0.8341	0.067*	
O1	0.4664 (4)	0.9701 (4)	0.59614 (16)	0.0626 (10)	
O2	0.4551 (4)	0.8193 (4)	0.43812 (17)	0.0717 (11)	
C1	0.8311 (6)	1.1218 (5)	0.5757 (2)	0.0521 (12)	
H1A	0.7493	1.0967	0.5367	0.063*	
C2	0.9839 (7)	1.1859 (6)	0.5539 (3)	0.0659 (15)	
H2A	1.0065	1.2022	0.4998	0.079*	
C3	1.1059 (7)	1.2271 (6)	0.6109 (3)	0.0740 (16)	
H3A	1.2082	1.2719	0.5946	0.089*	
C4	1.0774 (6)	1.2024 (6)	0.6922 (3)	0.0652 (15)	
H4A	1.1595	1.2291	0.7306	0.078*	
C5	0.9233 (6)	1.1369 (5)	0.7139 (3)	0.0523 (12)	
C6	0.7981 (6)	1.0942 (5)	0.6563 (2)	0.0441 (11)	
C7	0.6551 (6)	1.0313 (5)	0.6998 (2)	0.0452 (11)	
C8	0.6986 (6)	1.0394 (5)	0.7821 (3)	0.0520 (12)	
C9	0.5853 (7)	0.9866 (5)	0.8409 (2)	0.0595 (14)	
H9A	0.6131	0.9928	0.8953	0.071*	
C10	0.4327 (7)	0.9258 (6)	0.8160 (3)	0.0648 (15)	
H10A	0.3572	0.8893	0.8546	0.078*	
C11	0.3850 (7)	0.9158 (6)	0.7348 (3)	0.0651 (15)	
H11A	0.2803	0.8731	0.7198	0.078*	
C12	0.4970 (6)	0.9709 (5)	0.6776 (2)	0.0501 (12)	
C13	0.3242 (6)	0.8870 (6)	0.5679 (2)	0.0648 (15)	
H13A	0.2137	0.9217	0.5890	0.078*	
H13B	0.3387	0.7909	0.5849	0.078*	
C14	0.3258 (7)	0.8962 (7)	0.4805 (3)	0.0706 (15)	
H14A	0.3019	0.9894	0.4589	0.085*	
C15	0.2734 (6)	0.7819 (6)	0.4288 (3)	0.0687 (16)	
H15A	0.2331	0.6966	0.4541	0.082*	
H15B	0.2164	0.8046	0.3783	0.082*	

### Atomic displacement parameters (Å^2^)

**Table:**

	*U*^11^	*U*^22^	*U*^33^	*U*^12^	*U*^13^	*U*^23^
N1	0.057 (2)	0.073 (3)	0.0360 (19)	−0.006 (2)	−0.0127 (19)	−0.0015 (19)
O1	0.0521 (18)	0.092 (3)	0.0436 (17)	−0.012 (2)	−0.0062 (15)	−0.0092 (17)
O2	0.053 (2)	0.115 (3)	0.0468 (19)	−0.007 (2)	0.0064 (17)	−0.0009 (19)
C1	0.055 (3)	0.059 (3)	0.042 (2)	−0.002 (3)	−0.006 (2)	0.004 (2)
C2	0.068 (4)	0.090 (4)	0.040 (3)	−0.011 (4)	−0.003 (3)	0.005 (3)
C3	0.065 (3)	0.093 (4)	0.064 (3)	−0.025 (3)	0.002 (3)	−0.003 (3)
C4	0.061 (3)	0.080 (4)	0.055 (3)	−0.008 (3)	−0.012 (3)	−0.004 (3)
C5	0.053 (3)	0.061 (3)	0.043 (2)	0.000 (3)	−0.001 (2)	0.000 (2)
C6	0.045 (3)	0.050 (3)	0.037 (2)	0.004 (2)	−0.003 (2)	0.001 (2)
C7	0.058 (3)	0.042 (3)	0.035 (2)	0.007 (3)	−0.005 (2)	−0.004 (2)
C8	0.054 (3)	0.057 (3)	0.046 (2)	0.004 (3)	−0.005 (2)	−0.003 (2)
C9	0.071 (4)	0.070 (4)	0.037 (2)	0.005 (3)	0.004 (2)	0.002 (2)
C10	0.076 (4)	0.070 (4)	0.048 (3)	0.000 (3)	0.021 (3)	0.002 (3)
C11	0.059 (3)	0.079 (4)	0.058 (3)	−0.014 (3)	0.015 (3)	−0.005 (3)
C12	0.050 (3)	0.058 (3)	0.043 (2)	0.007 (3)	−0.004 (2)	−0.011 (2)
C13	0.045 (3)	0.092 (4)	0.057 (3)	−0.007 (3)	−0.002 (2)	−0.012 (3)
C14	0.058 (3)	0.093 (4)	0.060 (3)	−0.007 (3)	−0.011 (3)	−0.003 (3)
C15	0.056 (3)	0.084 (4)	0.066 (3)	−0.015 (3)	−0.003 (3)	−0.008 (3)

### Geometric parameters (Å, °)

**Table:**

N1—C8	1.363 (6)	C6—C7	1.439 (6)
N1—C5	1.389 (6)	C7—C12	1.386 (6)
N1—H1	0.8600	C7—C8	1.409 (5)
O1—C12	1.375 (4)	C8—C9	1.399 (6)
O1—C13	1.424 (5)	C9—C10	1.364 (7)
O2—C14	1.418 (6)	C9—H9A	0.9300
O2—C15	1.438 (6)	C10—C11	1.401 (6)
C1—C2	1.365 (7)	C10—H10A	0.9300
C1—C6	1.389 (5)	C11—C12	1.382 (6)
C1—H1A	0.9300	C11—H11A	0.9300
C2—C3	1.385 (6)	C13—C14	1.455 (6)
C2—H2A	0.9300	C13—H13A	0.9700
C3—C4	1.388 (6)	C13—H13B	0.9700
C3—H3A	0.9300	C14—C15	1.449 (7)
C4—C5	1.380 (6)	C14—H14A	0.9800
C4—H4A	0.9300	C15—H15A	0.9700
C5—C6	1.412 (6)	C15—H15B	0.9700
			
C8—N1—C5	110.0 (4)	C10—C9—H9A	121.1
C8—N1—H1	125.0	C8—C9—H9A	121.1
C5—N1—H1	125.0	C9—C10—C11	122.9 (5)
C12—O1—C13	117.2 (4)	C9—C10—H10A	118.6
C14—O2—C15	61.0 (3)	C11—C10—H10A	118.6
C2—C1—C6	119.7 (4)	C12—C11—C10	118.4 (5)
C2—C1—H1A	120.1	C12—C11—H11A	120.8
C6—C1—H1A	120.1	C10—C11—H11A	120.8
C1—C2—C3	121.2 (4)	O1—C12—C11	124.9 (4)
C1—C2—H2A	119.4	O1—C12—C7	114.3 (4)
C3—C2—H2A	119.4	C11—C12—C7	120.8 (4)
C2—C3—C4	120.8 (5)	O1—C13—C14	106.8 (4)
C2—C3—H3A	119.6	O1—C13—H13A	110.4
C4—C3—H3A	119.6	C14—C13—H13A	110.4
C5—C4—C3	117.8 (4)	O1—C13—H13B	110.4
C5—C4—H4A	121.1	C14—C13—H13B	110.4
C3—C4—H4A	121.1	H13A—C13—H13B	108.6
C4—C5—N1	130.4 (4)	O2—C14—C15	60.2 (3)
C4—C5—C6	121.9 (4)	O2—C14—C13	118.1 (5)
N1—C5—C6	107.7 (4)	C15—C14—C13	123.0 (5)
C1—C6—C5	118.5 (4)	O2—C14—H14A	114.8
C1—C6—C7	134.5 (4)	C15—C14—H14A	114.8
C5—C6—C7	106.9 (3)	C13—C14—H14A	114.8
C12—C7—C8	119.0 (4)	O2—C15—C14	58.8 (3)
C12—C7—C6	134.3 (4)	O2—C15—H15A	117.9
C8—C7—C6	106.7 (4)	C14—C15—H15A	117.9
N1—C8—C9	130.3 (4)	O2—C15—H15B	117.9
N1—C8—C7	108.7 (4)	C14—C15—H15B	117.9
C9—C8—C7	121.0 (4)	H15A—C15—H15B	115.0
C10—C9—C8	117.8 (4)		
			
C6—C1—C2—C3	−1.5 (8)	C6—C7—C8—N1	−0.9 (5)
C1—C2—C3—C4	1.0 (9)	C12—C7—C8—C9	0.5 (7)
C2—C3—C4—C5	−0.6 (9)	C6—C7—C8—C9	−180.0 (4)
C3—C4—C5—N1	−178.9 (5)	N1—C8—C9—C10	−178.0 (5)
C3—C4—C5—C6	0.7 (8)	C7—C8—C9—C10	0.7 (7)
C8—N1—C5—C4	179.0 (5)	C8—C9—C10—C11	−0.8 (8)
C8—N1—C5—C6	−0.7 (5)	C9—C10—C11—C12	−0.4 (8)
C2—C1—C6—C5	1.5 (7)	C13—O1—C12—C11	−10.7 (7)
C2—C1—C6—C7	179.4 (5)	C13—O1—C12—C7	168.4 (4)
C4—C5—C6—C1	−1.2 (7)	C10—C11—C12—O1	−179.3 (5)
N1—C5—C6—C1	178.5 (4)	C10—C11—C12—C7	1.7 (8)
C4—C5—C6—C7	−179.6 (4)	C8—C7—C12—O1	179.1 (4)
N1—C5—C6—C7	0.1 (5)	C6—C7—C12—O1	−0.3 (8)
C1—C6—C7—C12	1.9 (10)	C8—C7—C12—C11	−1.7 (8)
C5—C6—C7—C12	180.0 (5)	C6—C7—C12—C11	178.9 (5)
C1—C6—C7—C8	−177.6 (5)	C12—O1—C13—C14	−176.5 (4)
C5—C6—C7—C8	0.5 (5)	C15—O2—C14—C13	113.9 (6)
C5—N1—C8—C9	179.9 (5)	O1—C13—C14—O2	75.6 (6)
C5—N1—C8—C7	1.0 (5)	O1—C13—C14—C15	146.6 (5)
C12—C7—C8—N1	179.5 (4)	C13—C14—C15—O2	−106.0 (6)

### Hydrogen-bond geometry (Å, °)

**Table:**

*D*—H···*A*	*D*—H	H···*A*	*D*···*A*	*D*—H···*A*
N1—H1···O2^i^	0.86	2.09	2.948 (5)	172

Symmetry codes: (i) −*x*+3/2, −*y*+2, *z*+1/2.
